# Historical Roots of Research in Nanotechnology Application in Dental Composites: A Bibliometric Analysis: Historical Roots of Research in Nanotechnology Application in Dental Composites

**DOI:** 10.31661/gmj.v13iSP1.3725

**Published:** 2024-12-31

**Authors:** Sajad Raeisi Estabragh, Behzad Vosooghinezhad, Nader Ghotbi, Hamideh Soleimannejad, Pouya Sabanik, Ali Hashemzadeh, Zahra Lotfhaghpanah

**Affiliations:** ^1^ Department of Prosthodontics and Oral and Dental Diseases Research Center, Kerman University of Medical Sciences, Kerman, Iran; ^2^ Dentistry Department, European University, Tbilisi, Georgia; ^3^ Isfahan Azad University, School of Dentistry, Isfahan, Iran; ^4^ School of Dentistry, Tehran University of Medical Sciences, Tehran, Iran; ^5^ Department of Cariology, Restorative Sciences and Endodontics, School of Dentistry, University of Michigan, Ann Arbor, Michigan, USA; ^6^ Sri Rajiv Gandhi College of Dental Science and Hospital; ^7^ Department of Operative Dentistry, School of Dentistry, Shiraz University of Medical Sciences, Shiraz, Iran

**Keywords:** Dental Materials, Nanotechnology, Composite Resins, Antimicrobial Agents, Restorative Dentistry

## Abstract

**Background:**

The development of nanotechnology in dental composites has revolutionized the field of restorative dentistry. However, the historical roots of this field are not well understood.

**Materials and Methods:**

A bibliometric analysis was conducted using the Web of Science database to identify research roots in nanotechnology application in dental composites. A comprehensive search was performed using a specific search syntax, and 863 study data were exported. The data was analyzed using the Bibliometrix R package, and a historical direct citation network was employed to analyze the historical development of the research field.

**Results:**

The analysis revealed a moderate growth rate of 18.32% and a high degree of international collaboration in the field. The average age of documents was 6.12 years, with an average of 28.83 citations per document. The study identified key publications and authors that have shaped the development of the research field over time. The analysis highlighted the importance of concepts such as silanization, dual silanization, and interfacial phase reactivity in the development of dental nanocomposites. Additionally, the study found that the development of antimicrobial dental materials has been a significant area of research, with a focus on the use of quaternary ammonium polyethylenimine nanoparticles and silver nanoparticles to inhibit bacterial growth and biofilm formation.

**Conclusion:**

This study identified key publications and authors that have shaped the field and highlights the importance of concepts that have driven the development of dental nanocomposites, including antimicrobial properties. The findings of this study can inform future research and development in the field of dental nanocomposites.

## Introduction

The utilization of nanocoatings in dentistry is used in a diverse range of applications, including dental instruments, implants, components, and equipment. The presence of electrolytic fluids within the human body renders the internal environment highly corrosive and reactive, even for titanium and stainless-steel alloys [1][2][3][4].

Consequently, coatings with suitable mechanical properties and chemical neutrality are essential. Among the benefits of employing nanoparticles in dental restoration are the production of high-mesh fillers, minimal setting time, material stability and resistance to mechanical impacts, reduced tool adhesion, and precise color matching. The oral cavity presents a challenging environment, leading to concerns regarding the longevity of filled teeth [2][3][4].

Despite ongoing efforts, fillings may gradually fracture under various stresses, and over time, caries can develop at the interface between the filling and the tooth [3][4]. Nanotechnology holds the potential to mitigate these issues by producing dental restoratives that are more durable, stronger, and resistant to secondary decay compared to conventional fillers. The challenge with anti-caries composite fillers arises from an additive in the powder, where calcium and phosphate ions are incorporated for sustained release [5][6].

These ions are crucial for the longevity of the filling as they not only reinforce the tooth’s crystal structure but also safeguard it from the decay-causing acid produced by oral bacteria. However, the compounds responsible for ion release are structurally weak, compromising the filler’s integrity. To address this, a novel approach has been devised, reducing the particle size of these compounds, such as anhydrous dicalcium phosphate, to approximately 50 nm, which is 20 times smaller than the one-micron particles typically found in applied powders [7][8].

The high surface-to-volume ratio of these nanoparticles enhances their ion release efficiency, requiring significantly less material to achieve the desired effect. The calcium phosphate nanocomposite filling, when applied to a tooth, can intelligently release anti-caries agents that shield the tooth from acid-induced damage. Moreover, it can replenish lost minerals within the tooth’s ineffective mineralized areas by releasing ions, thereby enhancing the tooth’s overall health and durability.

## Materials and Methods

This was a bibliometric analysis to identify research roots in nanotechnology application in dental composites. To identify relevant studies on nano applications in dental resins, we conducted a comprehensive search in the Web of Science (WOS) database using the following search syntax:

TS=(nanoparticle* OR nano-particle* OR nano-composite*) AND TS=(resin-based OR polymer-based OR composite resin) AND TS=(dental OR tooth OR restorative)

This search query was designed to capture a broad range of studies related to nano applications in dental resins, including those that employed nanoparticles, nano-composites, or other nano-based materials in resin-based, polymer-based, or composite resin dental applications. The search was limited to studies that included keywords related to dental, tooth, or restorative applications. Total number of 863 study data were exported from WOS. Data was analyzed by Bibliometrix R package [9].

To analyze the historical development of the research field, we employed a novel approach using a historical direct citation network. This method, introduced by Garfield, represents a chronological network map of most relevant direct citations resulting from a bibliographic collection. We generated a historical citation network matrix using the histNetwork function, which was applied to our bibliographic collection. This resulted in a chronological direct citation network matrix, which was then plotted using the histPlot function with a node size of 10.

In addition, we applied Reference Publication Year Spectroscopy (RPYS) to detect the historical roots of the research field [10]. This method, introduced by Marx et al. (2014), analyzes the distribution of reference publication years to identify the most influential works in a field [10]. By applying RPYS, we aimed to identify the key publications that have shaped the development of the research field over time.

## Results

**Table:** Table1. Many Studies Used This Concept as Core of Their Innovation

**Study**	**Nanoparticle**	**Antibacterial Agent **	**Calcium Phosphate **	**Results**
**Cheng et al. (2012) [25] **	NACP (116 nm)	QADM	Present	Reduced biofilm CFU by 3-fold, metabolic activity, and lactic acid production
**Cheng et al. b(2012) [26] **	NACP	QADM, NAg	Present	Strongly antibacterial, reduced biofilm CFU, metabolic activity, and lactic acid production
**Melo et al. (2013) [27] **	NACP	QADM, NAg	Present	Novel dental adhesive with antibacterial agents and calcium phosphate nanoparticles
**Zhou et al. (2013) [28] **	NACP	DMADDM (new quaternary ammonium monomer)	Present	Potent anti-biofilm activity, reduced metabolic activity, lactic acid production, and biofilm CFU
**Cheng et al. (2012) [24] **	NAg (2.7 ± 0.6 nm)	QADM	-	Increased bacteria inhibition zone by 9-fold, reduced lactic acid production and CFU
**Zhang et al. (2013) [29] **	NAg, NACP	DMADDM	Present	No strength loss after 6 months of water-ageing, reduced biofilm viability and acid production
**Li et al. (2014) [30] **	-	DMAHDM (new quaternary ammonium methacrylate)	-	Increased quaternary amine charge density reduced bacteria early-attachment and biofilm CFU, without compromising bond strength

This bibliometric query analyzed a dataset spanning 25 years (1999-2024) and comprising 863 documents, 3221 authors, and 1814 unique author-assigned keywords. The field exhibited a moderate growth rate of 18.32% and a high degree of international collaboration, with 35.11% of documents resulting from co-authorships. The average age of documents was 6.12 years, with an average of 28.83 citations per document. Notably, 15 authors accounted for single-authored documents, and the top sources contributed 248 unique publications.

The top 10 most cited documents in this field were identified, with the most cited document being Yin et al. (2020) [2] with 899 citations, followed by Moszner et al. (2001) [2] with 522 citations, Beyth et al. (2006) [3] with 375 citations, Sevinç et al. (2010) [4] with 286 citations, Cheng et al. (2012) [5] with 263 citations, Chen et al. (2010) [6] with 249 citations, Xu et al. (2011) [7] with 223 citations, Ahn et al. (2009) [8] with 209 citations, Sadat-Shojai et al. (2010) with 200 citations, and Noronha et al. (2017) [9] with 188 citations.

In the early 2000s, a pivotal moment in the evolution of polymeric dental composites was marked by the publication of "New Developments of Polymeric Dental Composites" by Norbert Moszner and Ulrich Salz in 2001 [1]. This study highlighted the pioneering efforts of the time, which focused on overcoming the limitations of traditional composites by mitigating polymerization shrinkage, enhancing biocompatibility, and improving wear resistance and processing properties. The introduction of novel monomers, such as cyclic, liquid-crystalline, and branched monomers, was a groundbreaking achievement that paved the way for future innovations. Notably, the use of ring-opening polymerizable cyclic monomers was a significant breakthrough that showed promise in reducing polymerization shrinkage, thereby enhancing marginal adaptation and minimizing the risk of recurrent caries. Furthermore, the exploration of bioactive components in restorative materials, such as those found in composites like Heliomolar and Tetric ceram, was a notable trend in 2001, which aimed to promote remineralization and prevent demineralization of tooth structure [10]. As we look back, it's clear that these early advancements laid the foundation for the modern dental composites we use today. Prior to Moszner and Salz's 2001 study, the only notable research on nanoparticles in dental composites was Furman et al.'s 2000 study, "Metal-Oxide Nanoparticles for the Reinforcement of Dental Restorative Resins" [11], which explored the use of metal oxide nanoparticles to reinforce dental resins, although the results showed inferior mechanical properties compared to unfilled materials. In 2005, a seminal study by Wilson, Zhang, and Antonucci [12] marked a significant milestone in the evolution of dental nanocomposites, as they systematically investigated the impact of interfacial phase reactivity on critical composite properties. By silanizing silica nanoparticles with varying ratios of 3-methacryloxypropyltrimethoxysilane (MPTMS) and octyltrimethoxysilane (OTMS), the researchers demonstrated that dual silanization can improve the workability of composite pastes, particularly at high filler loadings. This breakthrough finding had far-reaching implications for the development of dental restorative materials, as it suggested that the strategic design of the interfacial phase could mitigate polymerization stress, reduce water sorption, and enhance the overall performance of nanocomposites. The study's results, which showed that dual silanized fillers with a blend of MPTMS and OTMS can achieve mechanical properties comparable to those of single-silanized fillers, paved the way for future research into the optimization of interfacial phase chemistry in dental nanocomposites. Hosseinalipour et al. (2010) [13] investigated the mechanical properties of dental composite resins containing various mass fractions of silica nanoparticles, building on the foundational work of Wilson, Zhang, and Antonucci (2005) [12], who pioneered the use of silica nanoparticles in dental nanocomposites and demonstrated the significance of interfacial phase reactivity on critical composite properties. Specifically, Hosseinalipour et al. (2010) [13] applied the concept of dual silanization, first introduced by Wilson et al. (2005), to modify the silica nanoparticles with γ-methacryloxy propyl trimethoxy silane (γ-MPS), which enhanced the filler-matrix interfacial bonding and contributed to the improved mechanical properties of the composites.

Rooted in the tradition of Moszner and Salz's 2001 study [1] and Wilson, Zhang, and Antonucci's 2005 study [12], Karabela and Sideridou's 2011 [14] study further advanced the field of dental nanocomposites. This study investigated the impact of nanosilica particle size on the physical and mechanical properties of light-cured composites, revealing that smaller particle sizes increase the degree of conversion and water sorption, while maintaining similar flexural strength and modulus. Later, Habib et al.'s 2013 [15] review article overviewed the evolution of dental resin composites, showing the importance of inorganic fillers and additives. Wang et al.'s 2015 and 2013 studies [16][17] further advanced the field by exploring the use of bimodal silica nanostructures as fillers, which improved the physical, mechanical, and wear properties of resin-based composites. While previously a 2006 study by Kim et al. [18] has had pioneered size control and surface treatment of silica nanoparticles for dental nanocomposites, laying groundwork for subsequent advancements achieved by Wang et al.'s [16][17]. Its findings on optimal surface treatment and dispersion improved adhesion and mechanical properties, influencing later research on bimodal silica nanostructures and high-performance dental materials, thereby enhancing the field's evolution. Finally, these advancements led to the development of novel nanofibrous fillers, such as hydroxyapatite (HA) nanowires, that can provide both efficient reinforcement and high antibacterial activity. A study published in 2017 by Ai et al. [19] demonstrated the successful synthesis of HA nanowires via hydrothermal technique, followed by surface modification with mussel-inspired dopamine (DA) and loading of silver nanoparticles (AgNPs). The resulting composite resins showed improved mechanical properties, homogeneous distribution of AgNPs, and high antibacterial activity against streptococcus mutans. This study builds upon the foundational work of researchers such as Moszner and Salz (2001), Wilson, Zhang, and Antonucci (2005), and Karabela and Sideridou (2011) [14], who explored the use of nanoparticles and interfacial phase reactivity to enhance the properties of dental composites.

**Figure-1:**
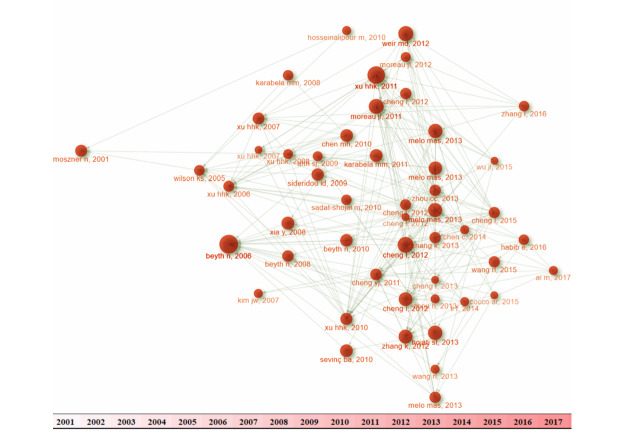
Figure 1. Figure 1 shows the historiography of the study

**Figure-2:**
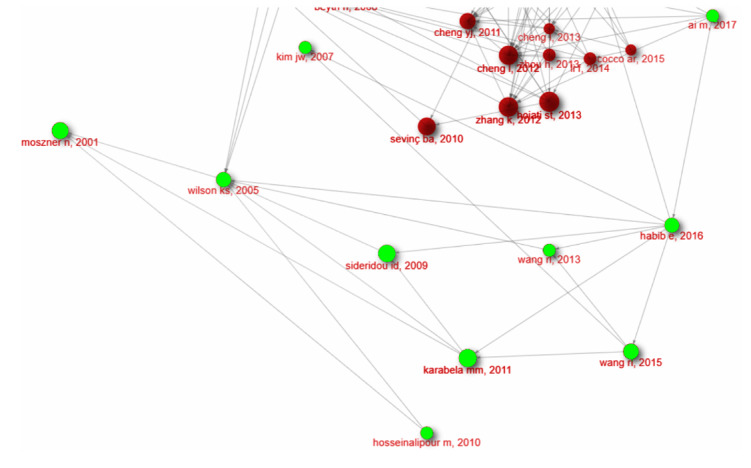
Figure 2. This chronological chain of evidence led highlighting importance of concepts of silanization, Dual silanization, Interfacial phase reactivity

**Figure-3:**
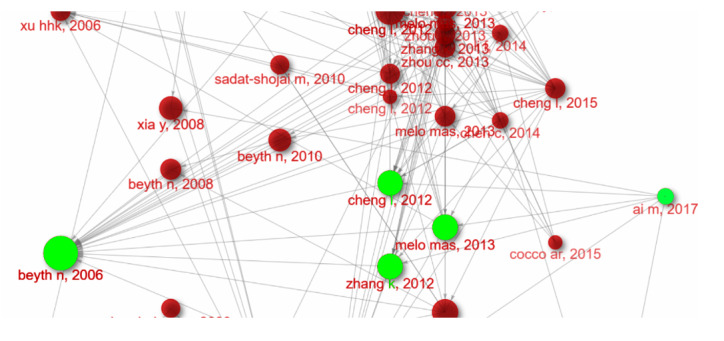
Figure 3. Chronological chain of evidence for QADM+ Nag application

But in a smaller subnetwork, the concepts of silanization were also emphasized. Expanding on the ideas of Wilson et al. in 2005, Karabela and Sideridou [20] investigated the specific role of silane structure in modulating water and ethanol/water sorption behavior. Their research revealed that the silane structure, particularly the use of urethane dimethacrylate silane (UDMS), octyltrimethoxysilane (OTMS), and blends thereof, significantly influenced the sorption characteristics of dental nanocomposites. Later, a review by Chen [21] in 2010 provided a timely update on the latest developments and clinical applications of these materials.

Chen's review highlighted the diverse range of modifications being made to the three main components of dental nanocomposites: inorganic fillers, organic resin matrix, and silane coupling agents. The review also touched on the importance of silanization and its effects on the properties of nanocomposites, as well as clinical considerations, including light-curing modes and mechanical properties. Building on the advancements made in the early 2000s, particularly the breakthrough findings of Wilson, Zhang, and Antonucci (2005) on the strategic design of the interfacial phase in dental nanocomposites, researchers continued to explore innovative approaches to overcome the limitations of traditional composites. In 2007, a significant milestone was achieved by Xu, Weir, and Sun [22], who synthesized nanoparticles of dicalcium phosphate anhydrous (DCPA) and incorporated them into a dental resin, aiming to create a mechanically strong composite that released calcium and phosphate ions to combat tooth caries. The researchers' findings showed that decreasing the DCPA particle size increased the release of calcium and phosphate ions, while whisker reinforcement significantly enhanced the composite's strength and elastic modulus. Moreover, silanization of the DCPA particles improved the composite's strength but reduced the ion release.

One of most node sized studies in this investigation was Beyth et al. [3] study in 2006. They embedded quaternary ammonium polyethylenimine nanoparticles into dental composites, which showed potent antibacterial activity against Streptococcus mutans. The nanoparticles demonstrated long-lasting efficacy without compromising the mechanical properties of the composites. Fast forward to 2012, when two significant studies were published. Zhang et al. [23] investigated the effect of incorporating quaternary ammonium dimethacrylate (QADM) and nanoparticles of silver (NAg) into dental adhesives and primers. Their findings showed that these antibacterial agents could be successfully integrated into dental materials without compromising dentin bond strength, paving the way for the development of antibacterial dental restoratives. Around the same time, Cheng et al. [24] published a study on the development of an anti-biofilm dentin primer containing QADM and NAg, which demonstrated potent antibacterial activity against dental plaque microcosm biofilms. In 2017, Ai et al. [18] built upon the earlier research by demonstrating the successful synthesis of HA nanowires and their surface modification with mussel-inspired dopamine and loading of silver nanoparticles. This study further expanded the possibilities for antibacterial dental materials, highlighting the potential of nanotechnology in the development of novel antibacterial agents.

Later, many studies used this concept as core of their innovation as shown in Table-1.

## Conclusion

The study has mapped the growth and evolution of the field, highlighting key milestones, influential authors, and pivotal concepts that have driven innovation. The findings underscore the significance of silanization, dual silanization, and interfacial phase reactivity in the development of dental nanocomposites, as well as the growing importance of antimicrobial properties in preventing bacterial growth and biofilm formation.

## Conflict of Interest

None.
